# The role of ABC transporter DrrABC in the export of PDIM in *Mycobacterium tuberculosis*

**DOI:** 10.1016/j.tcsw.2024.100132

**Published:** 2024-10-15

**Authors:** Nabiela Moolla, Helen Weaver, Rebeca Bailo, Albel Singh, Vassiliy N. Bavro, Apoorva Bhatt

**Affiliations:** a School of Biosciences and Institute of Microbiology and Infection, University of Birmingham, Edgbaston, Birmingham B15 2TT, United Kingdom; b School of Life Sciences, University of Essex, Colchester CO4 3SQ, United Kingdom

## Abstract

The *Mycobacterium tuberculosis* virulence lipid phthiocerol dimycocerosate (PDIM) is exported by a complex mechanism that involves multiple proteins including the Resistance-Nodulation-Division (RND) transporter MmpL7 and the lipoprotein LppX. Here, we probe the role of the putative heterooligomeric ATP-Binding Cassette (ABC) transporter complex composed of DrrA, DrrB and DrrC in PDIM transport by constructing a set of individual null mutants of *drrA*, *drrB* and *drrC* in the vaccine strain *Mycobacterium bovis* BCG. Loss of all three, or individual *drr* genes, all resulted in a complete loss of PDIM export to the outer envelope of the mycobacterial cell. Furthermore, guided by a bioinformatic analysis we interrogated specific signature residues within the DrrABC to demonstrate that it is indeed an ABC transporter, and our modelling, together with the mutagenesis identify it as a member of the Type V family of ABC exporters. We identify several unique structural elements of the transporter, including a non-canonical C-terminally inserted domain (CTD) structure within DrrA, which may account for its functional properties.

## 1 Introduction

**Fig. 1:**
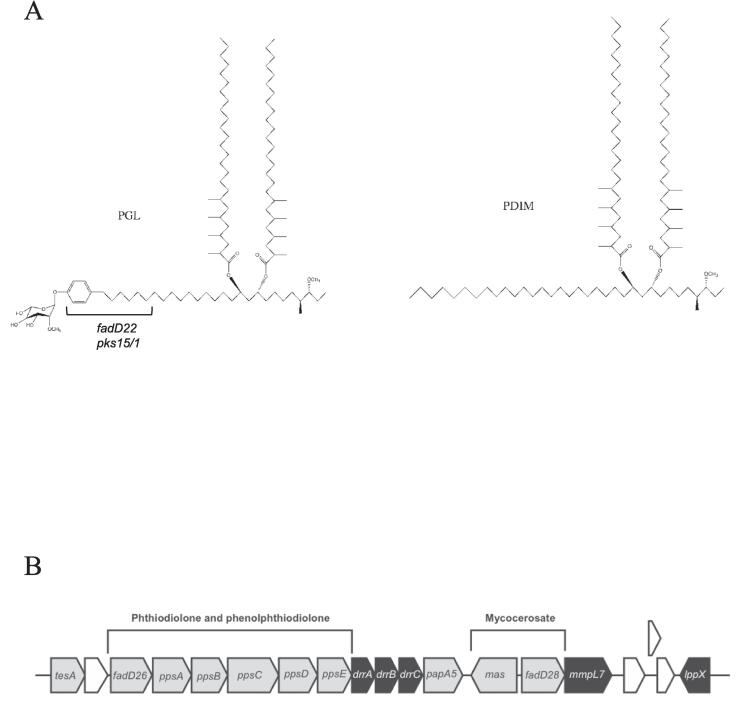
(A) Structures of phenolic glycolipid (PGL) and phthiocerol dimycocerosate (PDIM), and (B) genome organisation of the PDIM/PGL biosynthesis cluster in *M. tuberculosis/M. bovis*. In *M. tuberculosis* H37Rv, the *pks1/15* gene is mutated due to a frameshift.

The waxy cell wall of *Mycobacterium tuberculosis*, the causative agent of tuberculosis, is composed of an outer envelope containing an assortment of surface exposed lipids (Batt et al. 2020). Included in this outer layer are two key virulence lipids, phthiocerol dimycocerosates (PDIMs) and glycosylated phenol phthiocerol dimycocerosates or phenolic glycolipids (PGLs). PDIMs consist of a diol backbone (phthiocerol), esterified to two methyl-branched fatty acyl moieties (mycocerosic acid) (Fig. 1A). Phthiocerol is synthesised by a set of five Type-I polyketide synthases, encoded by *ppsA-ppsE*, while mycocerosic acid is produced by a Type-I fatty acid synthase termed mycocerosic acid synthase encoded by *mas* (Fig. 1B). PGLs share this common lipidic core, but are further elaborated by a phenolic group, and glycosylation, introduced by a polyketide synthase encoded by *pks1-15* and numerous glycosyl transferases (Constant et al. 2002). Both lipids are also found in other members of the *M. tuberculosis* complex including *Mycobacterium bovis*, and in other related pathogenic mycobacteria including *Mycobacterium leprae*, *Mycobacterium marinum* and *Mycobacterium kansasii* (Constant et al. 2002).

These phthiocerol-derived lipids are drivers of virulence and PDIM has been associated with macrophage invasion, disrupting phagosomal acidification and maturation, masking of pathogen associated molecular patterns (PAMPS), resistance to killing by nitric oxide, arresting the recruitment of activated macrophages to the site of infection, and more recently, inducing autophagy for phagosomal escape with the aid of the ESAT-6 secretion system (ESX-1) and the type I interferon response (Camacho et al., 2001, Cox et al., 1999, Rens et al., 2021). PGLs are associated with modulation of infected macrophages to suppress inflammatory immune responses by interfering with antigen-presenting cell function and decreasing nitric oxide required for killing. PGLs and PDIMs also act synergistically in *M. marinum* to help evade killing by the immune system and promote long term survival in the host (Cambier et al. 2014).

The transport of PDIMs to the outer envelope has been studied in some detail in *M. tuberculosis*. MmpL7, a member of the Resistance-Nodulation-Division (RND) family of membrane proteins plays a critical role in this process. Mutants of MmpL7 accumulate PDIM intracellularly (Camacho et al., 2001, Cox et al., 1999) and are also attenuated in animal models of infection, indicating the requirement for surface located PDIMs for virulence. Furthermore, two extracellular loops, connecting transmembrane helices 1 and 2, and 7 and 8 respectively, were shown to be critical for transport, and demonstrated to interact with late-stage biosynthetic enzymes, suggesting coupled biosynthesis and transport of PDIMs involving MmpL7 (Jain and Cox 2005). However, MmpL7 is not the sole transporter responsible for PDIM translocation. A null mutant of *lppX*, another gene belonging to the PDIM cluster and encoding a lipoprotein, was also found to be required for PDIM translocation leading to the suggestion that LppX may play a stabilizing role in the localisation of PDIMs to the surface (Sulzenbacher et al. 2006). The PDIM cluster also encodes a set of three proteins similar to ABC transporters: DrrA, DrrB and DrrC (Fig. 1B). Transposon insertions in *drrC* abolished PDIM transport, highlighting a role in PDIM transport (Camacho et al. 2001). These findings suggest that the transport of PDIMs may be a complex process driven by a multiprotein assembly that includes, but is not limited to MmpL7.

The origins of the annotation of the *M. tuberculosis drr* genes lay in their similarity to ABC exporters of the antibiotic daunorubicin in *Streptomyces peucetius,* where ‘*drr’* stands for daunorubicin resistance (Guilfoile and Hutchinson 1991). In *S. peucetius*, a daunorubicin producer, the DrrA and DrrB transporters serve to efflux the antibiotic, protecting the producer strain from its effects. Biochemical characterisation of *M. tuberculosis* DrrA and DrrB has shown that the two proteins constitute a functional pair, with the former binding ATP, while the latter forms an integral membrane conduit (Gandlur et al., 2004, Kaur and Russell, 1998). The function of DrrC is thought to be analogous to that of DrrB, as it is also predicted to be an integral membrane protein. Previous studies have shown that transposon mutants of *M. tuberculosis drrA*, and *drrC* lacked surface PDIMs (Camacho et al., 2001, Day et al., 2014). However, these studies do not account for the operonic nature of the *drrABC* genes and potential polar effects of the transposon insertions. Thus, a systematic study of precise, individual of *drrA*, *drrB* and *drrC* null mutants is warranted. It is also unclear whether they form a complex or operate individually to carry out PDIM transport. Here, we investigated the individual roles of *drrA*, *drrB* and *drrC* in PDIM transport and explored any redundant functions therein, by generating and characterising individual null mutants of these genes in the vaccine strain, *M. bovis* BCG, that unlike *M. tuberculosis* H37Rv produces both PDIMs and PGLs. Furthermore, we used structural insights provided from the homology modelling to identify functionally important motifs of the DrrABC transporter, allowing it to be firmly classified within the type V exporter ABC family, and to suggest the functional requirement for coupling between the nucleotide binding domains (NBDs) of DrrA and the porter subunits provided by DrrB and DrrC. In addition, we provide an *in-silico* description of the Drr-complex, and describe and demonstrate, for the first time, the functional requirement of the non-canonical C-terminal domain of DrrA for PDIM export.

## 2 Results

### 2.1 Construction of individual *drr* gene null mutants in *Mycobacterium bovis* BCG

**Fig. 2:**
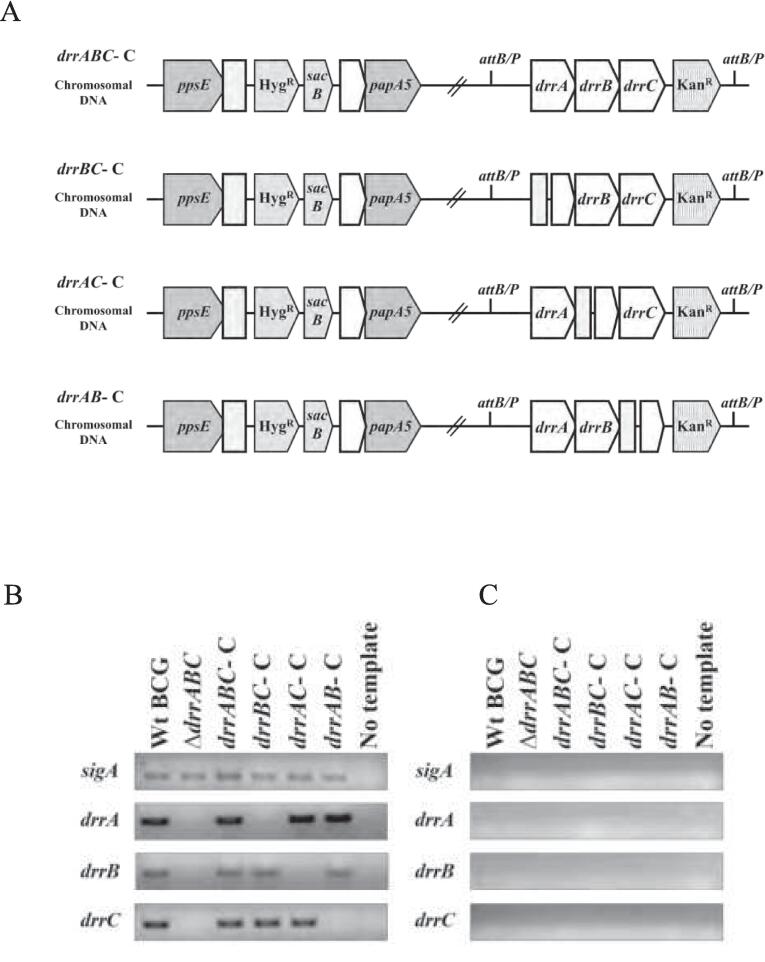
(A) Genomic organisation of recombinant M. bovis BCG mutant strains (B,C) RT-PCR analysis of *drrA*, *drrB* and *drrC* expression in associated *M. bovis* BCG mutant strains. (B) expression of individual *drr* genes in the wild type (Wt) BCG, the Δ*drrABC* mutant and the four complemented strains as compared to the expression of the housekeeping gene *sigA* as a positive control. (C) depicts the same reactions carried out in the absence of reverse transcriptase as a control.

We selected *Mycobacterium bovis* BCG as a surrogate to study the potential role of *drrABC* in PDIM transport. As the *drr* genes form part of a predicted operon, to systematically study the loss of individual *drr* genes, we first simultaneously deleted *drrABC* using specialized transduction (Bardarov et al., 2002, Larsen et al., 2007) replacing the three genes with a hygromycin resistance cassette (*hyg*) to generate the triple deletion mutant strain BCG Δ*drrABC*. This strain was then transformed with an integrative plasmid containing *drrABC* with the associated native promoter, or with versions of this plasmid containing in-frame deletions in either *drrA*, *drrB* or *drrC*. The in-frame deletion in the complementing plasmids was expected to produce truncated, non-functional DrrA, DrrB or DrrC. Thus, the BCG Δ*drrABC* strain transformed with either of these recombinant plasmids represented individual *drrA*; *drrB,* or *drrC* null mutants respectively. The strains were accordingly designated BCG Δ*drrA*; Δ*drrB,* and Δ*drrC* (Fig. 2A) Expression of each intact (full-length) gene in the recombinant strains was confirmed by RT-PCR (Fig. 2).

### 2.2 Analysis of PDIM transport in BCG *drr* mutant strains

**Fig. 3:**
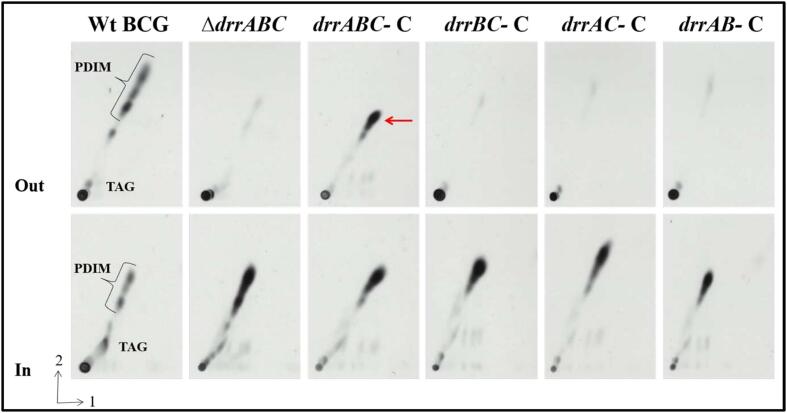
2D-TLC analysis [^14^C]-propionate labelled lipids extracted from *drr* mutant strains. Autoradiography of [^14^C]-propionate labelled lipids from Wild type (Wt) and mutant strains. Apolar lipids were separated using Systems A (Direction 1, petroleum ether (60–80 °C): ethyl acetate, 95:2 (v/v) X 3; Direction 2, Petroleum ether (60–80 °C): acetone 92:8 (v/v). System C: direction 1, chloroform: methanol 96:4 (v/v), direction 2, Toluene: acetone, 80:20 (v/v)).PDIM species are shown by a bracket. ‘Out’ and ‘In’ indicate exported and intracellular lipid fractions respectively. The red arrow indicates restoration of PDIM export in the complemented strain. (For interpretation of the references to colour in this figure legend, the reader is referred to the web version of this article.)

To assess the impact of null mutations in *drrA*, *drrB* and *drrC*, we analysed the export of PDIMs by the different mutant BCG strains by extracting [^14^C]-labelled lipids from bacterial cells and culture supernatants. Cells were labelled using [^14^C]-propionate (for labelling methyl branched fatty acid containing lipids including PDIMs). From previous reports we expected the *drrA* strain to be deficient in PDIM export. 2D-TLC analysis of the extracted [^14^C]-labelled lipids showed the loss of PDIMs in culture supernatants of the Δ*drrABC* strain (Fig. 3). PDIMs were present intracellularly, confirming that this was a transport defect rather than a loss of biosynthesis. Interestingly, the Δ*drrA*, Δ*drrB* and Δ*drrC* strains all showed a phenotype that was identical to the triple mutant, *i.e* loss of PDIM transport, indicating a non-redundant role for *drrA*, *drrB* and *drrC* in PDIM export.

### 2.3 Predicted structure of DrrA, DrrB and DrrC

**Fig. 4:**
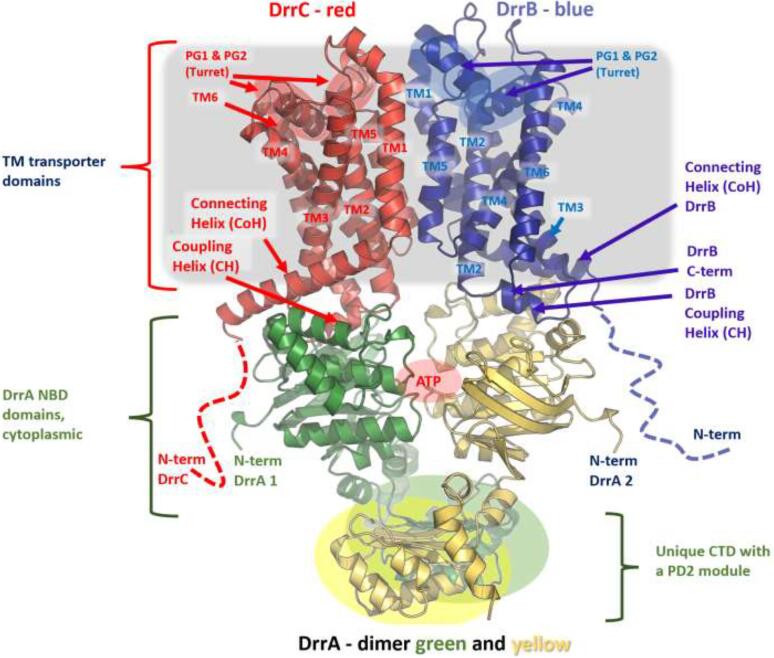
Predicted structural organisation of the assembled DrrABC_2_ complex based on the AlphaFold2 homology models. Individual subunits are coloured in red (DrrC), blue (DrrB), and green and yellow (for each of the DrrA monomers) respectively. The N-termini of DrrB and DrrC are predicted to be low complexity regions and are truncated at R25 (DrrB) and P16 (DrrC) respectively. Structural elements discussed in the text are highlighted. The position of the ATP-binding site between the two DrrA NBDs is highlighted by a red circle with an ATP sign. Approximate membrane position is indicated by a grey rectangle. (For interpretation of the references to colour in this figure legend, the reader is referred to the web version of this article.)

As there is no experimental structure of the DrrABC transporter or any of its close homologues currently available, to facilitate identification of potential, functionally important residues, we used homology modelling to create 3D models of DrrA, DrrB and DrrC using I-TASSER (Yang et al. 2015), alongside Swiss-Model (Waterhouse et al. 2018) in template-specific mode. After the main body of this work was completed, AlphaFold2 (AF2) (Jumper et al. 2021) became available, and due to its higher overall quality and public availability of the AF2 models, we have updated our analysis based on them (Fig. 4). For completeness, we also provide comparisons of the AF2-generated models with our initial 3D-models (Supplementary Fig. S1) and the full original template analysis and modelling is provided in the Supplementary Materials section.

Analysis of these models revealed important structural features of DrrABC, positioning it close to the recently defined Type V floppases/exporters (Thomas et al. 2020), which also include the Wzm-Wzt family of O-antigen exporters, which may indicate some similarity in function between these transporters and DrrABC (Supplementary Figure S2). The identified homology between the Wzm-Wzt and DrrABC, allowed us to reconstruct the complete assembly of the latter based on the former.

The general architecture of the DrrABC complex is suggested to be DrrBC-A_2_ (Fig. 4), with a heterodimer of DrrBC forming the transmembrane porter region, while the DrrA contains a classical nucleotide binding domain (NBD), which forms a cytoplasmic dimer supposed to provide the energy for cargo transport (Supplementary Figures S3-S4).

DrrA shows clear affiliation with ABC transporters, presenting both classical Walker A and Walker B motifs, as well as a conserved *H-loop* (Supplementary Figures S5-S6). It must be noted however, that the ABC-signature sequence (aka C-motif), which in most ABC transporters follows the canonical “LSGGQ” motif is not strictly conserved in the DrrA, and presents a rather hydrophobic element in the form of M145, followed by charged cluster of three arginine residues (RRR 146–148), giving it a unique signature (T_140_YSGGMRRR_148_), which is not typical of classic ABC-transporters, but rather resembling the modified signature seen in the O-antigen associated Wzm-Wzt family of ABC-transporters (e.g. in the WztN of *Aquifex aeolicus*, the corresponding sequence is TYSSGMIMRLAF) (Bi et al., 2018, Spellmon et al., 2022) (Supplementary Figures S5-S6). While this feature may appear to align DrrA with the Wzm-Wzt transporters and thus might suggest similarity of function, there are a number of discrepancies and unique features that separate it from that family. Notably, the sequence of the *Q-loop* in DrrA – located between the RecA-like subdomain and the α-helical subdomain – contains the two glutamine residues that give its name (G_88_QQVAVDD_95_), which are absent in the Q-loop of the O-antigen transporters (Supplementary Figures S5-S6), and furthermore, DrrA lacks the helical insertion after β1-strand of the RecA-subdomain (which corresponds to the loop *K*10-L34 in Wzm-WztN as seen in 6OIH.pdb) (Bi et al. 2018). In addition, the NBDs of DrrA contain a unique C-terminally inserted domain (CTD) structure (covering residues from D233 to the C-terminal R331), showing very low sequence similarity to known sequences, clearly standing out from other known NBD-domains (Fig. 4; Supplementary Figures S2–S3). While the role of this CTD is difficult to ascertain with certainty, our modelling suggests a remote structural homology to the Porter Domain (PD) modules found in the MmpLs and RND transporters (Alav et al., 2021, Moolla et al., 2021), and it is notable that the Wzm-WztN (8DKU.pdb)(Spellmon et al. 2022), also features a C-terminal NBD domain insertion, designated C-terminal carbohydrate-binding domain (CBD), and that corresponding domain is involved in substrate (in that case carbohydrate) binding. Extending the analogy, similar to the Wzm-WztN, DrrA also possesses what could be designated as hinge helix (Spellmon et al. 2022), that allows the CBD to couple to the NDB motions. We provide a side-by-side comparison of Wzm-WztN with DrrABC model to highlight their structural analogy (Supplementary Figure S2). The extreme C-terminal tail of DrrA (S306-R331) appears to be flexible and may be folding onto the rest of the NBD, or as can be seen in Wzm-WztN (8DKU.pdb), may contact the opposite NBD providing allosteric signalling (Spellmon et al. 2022).

DrrB and DrrC subunits forming the transmembrane porter section of the transporter are closely related by sequence (identity of 29 % and similarity of 43 % as defined by Ident and Sim programs (PMID: 10868275); Fig. 4), but present limited homology to other transporters of known structure, with the *blastp* algorithm producing no statistically significant matches against the PDB database. Nonetheless, sequence threading again identified several group V ABC-exporters as their closest relatives (including the aforementioned Wzm from *Aquifex aeolicus*; UniprotKB O67182 with identity 18.03 %; similarity 38.69 %), with a possible remote connection to the recently-defined MlaEF transporters, involved in maintaining the outer membrane phospholipid asymmetry (group VIII transporters) (Coudray et al., 2020, Tang et al., 2021). Consistent with the above, modelling of the DrrB and DrrC revealed a common fold and general topology consistent with Type V ABC transporters (Fig. 4; Supplementary Figure S2, Supplementary Figure S7), namely, comprising of 6 transmembrane (TM) helices, N- and C-termini in the cytoplasm, as well as a couple of specialized elements – notably the N-terminal Connecting Helices (CoH) and the Coupling helices (CH), the latter spliced between TM2 and TM3, which are suggested to couple the conformational changes of the NBDs upon ATP binding to outward opening of the TM channel, as seen in the Wzm (PDBID 6OIH; 6 M96) (Bi et al. 2018). There is also a notable extracellular “turret” formation, composed of a short 3_10_ helix, plus the short reentrant helices PG1 and PG2 (from periplasmic gating 1 and periplasmic gating 2), spliced between TM5 and TM6, which is also shared with Wzm (Caffalette & Zimmer 2021) (Fig. 4; Supplementary Fig S2; Supplementary Figure S7). The presence of CoH and CH structural elements can be seen not only in O-antigen transporters, but also within the MacAB and MlaEF family, and they are thought to be essential for the coupling of the ATP hydrolysis to the allosteric transitions leading to opening of the translocase (Coudray et al., 2020, Crow et al., 2017). Comparative analysis of the CH-residues of DrrB and DrrC with these of Wzm reveals conserved homologous residues (R121, F122 and P126 in DrrB and R113, W115, and P118 in DrrC respectively) (Supplementary Fig. S2; Supplementary Fig. S7), which further suggest affiliation of the DrrB/C with Type V exporters. Specifically, the bulky hydrophobic residues (F122 and W115, in DrrB and DrrC respectively) correspond to F90 in the Wzm-homodimer; R121/R113 to K89, while the prolines cap the helix (P96 in Wzm), as can be seen in e.g. 7KT2.PDB and 6 M96.PDB. Based on these predicted functional similarities we also set out to disrupt these key CH-residues to validate whether DrrBC utilizes a similar coupling mechanism to that of known type V exporters.

### 2.4 Mutational analysis of select residues in *drrA*, *drrB* and *drrC*

The construction of the Δ*drrABC* strain provides a capable platform for functional testing and we set out to test the role of select residues predicted by our *in silico* models to play an role in DrrA, DrrB or DrrC function. As a proof-of-principle for our mutagenesis platform we introduced the following mutants: within DrrA we targeted M145 (M145A) belonging to the unusual ABC-signature motif (*C-motif*) of DrrA (T_140_YSGGMRRR_148_), where it is predicted to participate in ATP-coordination (See Supplementary Fig. S5). In addition, intrigued by the unusual CTD seen in the DrrA, which has no analogue and has not been implicated in function, we created two deletions: ΔA224-R331, covering the whole of the C-terminal region, and ΔG232-P305, covering the predicted PD-like domain only (Supplementary Figure S4).

**Fig. 5:**
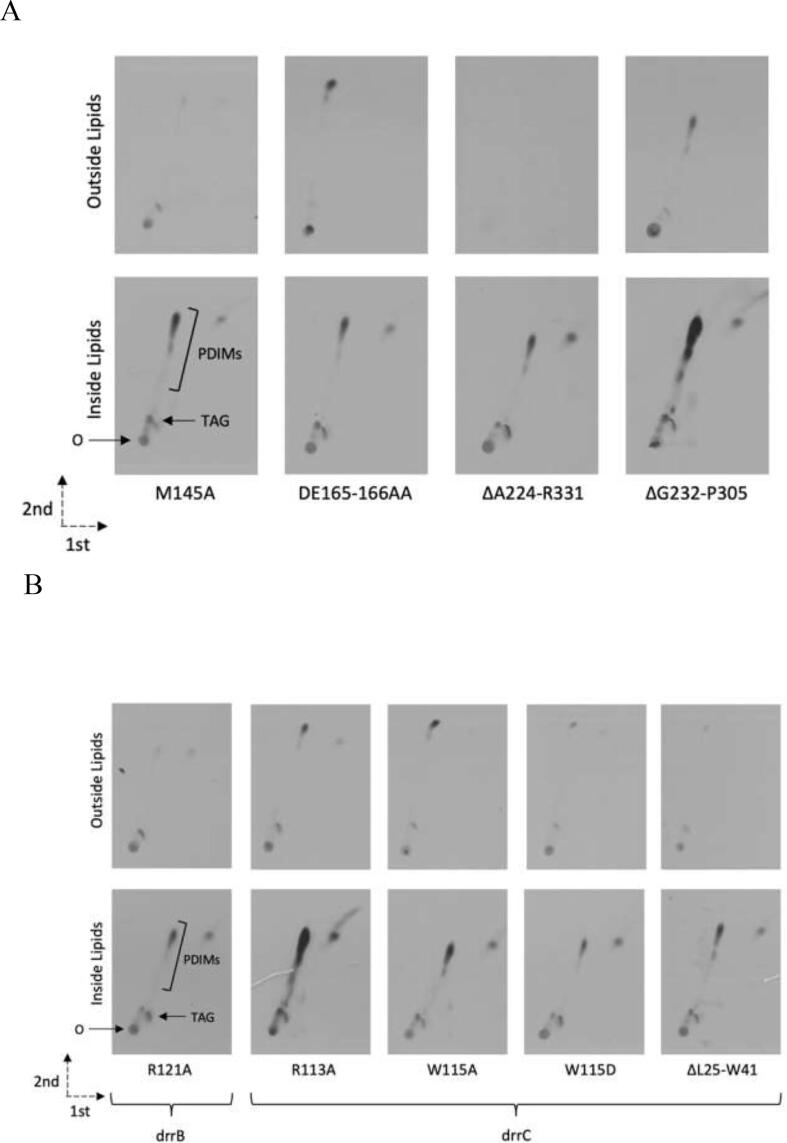
2D-TLC analysis [^14^C]-propionate labelled lipids extracted from the Δ*drrABC* strain complemented with mutated alleles of *drrA* (A), *drrB* or *drrC* (B). Apolar lipids were separated using System A (Direction 1, petroleum ether (60–80 °C): ethyl acetate, 95:2 (v/v) X 3; Direction 2, Petroleum ether (60–80 °C): acetone 92:8 (v/v)). ‘Out’ and ‘In’ indicate exported and intracellular lipid fractions respectively.

Using site-directed mutagenesis, we generated the above-mentioned mutated alleles of *drrA* on the complementing plasmid pMV306-*drrABC* and introduced them into the Δ*drrABC* strain. The transformed strains carrying a mutated allele of *drrA* and wild type copies of *drrB* and *drrC,* were labelled with ^14^[C] propionate and extracted lipid were analysed by 2D TLC to visualise PDIMS in cells and in culture supernatants, assessing the ability of the mutated alleles to complement the Δ*drrABC* strain. The C-motif associated M145 residue was required for function as the M145A mutant failed to restore PDIM transport in the Δ*drrABC* strain (Fig. 5). Furthermore, consistent with our hypothesis that the CTD-extension is directly involved in the substrate binding, deleting the region spanning A224-R331 covering the entire CTD also resulted in a loss of function, as no PDIM was detected in culture supernatants.

However, a partial deletion of the CTD, ΔG232-P305 (covering the predicted PD-like domain), led to a partial restoration of PDIM export, although accompanied with an accumulation of intracellular PDIMs (Fig. 5A; Supplementary Figure S4). Surprisingly, the DE165-166AA substitution did not seem to affect PDIM export.

Next, we targeted the conserved residues of the coupling helices (CH) in DrrB (R121) and in DrrC (W115) (Supplementary Figures S2; S7) which, as their name suggests are involved in coupling the conformational changes in the NBDs of the related transporter to the opening of their transmembrane porter domains. The coupling-helix mutation in DrrB (*drrB*_R121A) resulted in a loss of function as assessed by the ability to restore PDIM transport (Fig. 5B), while the one targeting the coupling-helix in DrrC (R113A) had a less drastic effect, with PDIM transport being partially restored, albeit with a larger proportion of intracellular PDIM accumulation. Additionally, deleting part of the connecting-helix (CoH), ΔL25-W45, resulted in the loss of ability to complement the mutant (Fig. 5B; see also Supplementary Figure S7).

The above results are consistent with DrrABC operating like a type V ABC exporter and suggest that the CTD plays a role in substrate binding and facilitating substrate insertion into the transmembrane channel of the transporter.

## 3 Discussion

### 3.1 DrrA, DrrB and DrrC are individually required for PDIM export

The loss of PDIM export in single null mutants of *drrA*, *drrB* or *drrC* suggested a critical role for each gene in the transport of this virulence lipid. Given that the *mmpL7* mutant has an identical phenotype (Camacho et al., 2001, Cox et al., 1999), and that MmpL7 is missing the canonical RND transporter residues forming the proton relay for utilising proton motive force (PMF) (Moola et al., 2021), and while there is yet no direct evidence, due to their functional linkage it is likely that MmpL7 provides a scaffold with DrrABC and facilitates the transport of PDIM.

### 3.2 Structural features of Drr-transporters suggest close affiliation with group v ABC exporters family and reveal a unique C-terminal domain with a substrate binding function

Both sequence and structural comparisons clearly identify the DrrABC as a member of the wider ABC transporter group, which is supported by the presence of the classical structural elements, including canonical Walker A and Walker B motifs, conserved H-loops and Q-loops within DrrA (Supplementary Figures S5-S6). Our structural modelling of DrrABC helps to clarify its evolutionary standing and provides suggestions about its function.

As mentioned above, numerous elements of the structure of DrrABC suggest close affiliation to the type V ABC-transporters (exporters) as their closest relatives (including the aforementioned Wzm-WztN O-antigen transporter) (Spellmon et al., 2022, Thomas et al., 2020). In particular, the modified ABC-signature, featuring a hydrophobic motif (represented by M145) highlights the relationship with the O-antigen ABC-transporters, where it consists of the sequence Y_142_-S-S/T-G-M−X−X−R/K-L-A/G-F_152_, of which S143 contacts ATP’s γ-phosphate and M146 packs into a hydrophobic pocket formed by Y142 and F134 (WztN numbering) (Supplementary Figures S5-S6). Consistently, the M145A mutant showed a loss of transport function demonstrating that preservation of this unusual ABC-motif is required for the function of the DrrABC transporter. We provide a comparison of the ATP binding pockets of DrrA, WztN and the ABC transporter Tm_1403, from *Thermotoga maritima* which is the closest homologue of DrrA of known structure, highlighting the discussed residues (Supplementary figure S5). Comparison of the complete predicted DrrABC assembly next to the experimentally determined, substrate-bound structure of Wzm-WztN is also provided as a Supplementary figure S2.

Despite the above similarities to type V transporters, the DrrABC also displays some unique features, including the unusual CTDs seen in DrrA. While lack of reliable template hindered our modelling, and similarly AlphaFold did not provide high confidence scores for these regions, the secondary structure suggests a possibility of a PD-like domain being spliced in there. The functional requirement for these domains is unclear and to date there have been no reports linking them to Drr function, hence we decided to truncate the C-terminus of DrrA from A224-R331, or use a more subtle deletion, removing only the predicted PD-like module (ΔG232-P305) (Supplementary Figure S4). Notably, both deletions severely impacted PDIM export, which for the first time demonstrates the functional importance of these unique structural features of this transporter. While the CTD observed in DrrA does not share direct homology with the CBD of Wzm-WztN (Spellmon et al. 2022), it is notable that both C-terminal extensions are spliced in equivalent positions and are predicted to be similarly coupled, using hinge helices, suggesting a common mode of allosteric action. Given that the CBD of Wzm-WztN directly binds the substrate and also provides allosteric coupling to NBD motion during the ATPase cycle, we propose that the CTD of DrrA acts in a similar manner. The fact, that the deletion of the PD-like module alone (ΔG232-P305) did not obliterate the function, but resulted in increased intracellular accumulation of PDIMs, suggests that the role of the PD-module within CTD may be to increase the local concentration of the substrate, but that it is not essential, while the rest of the CTD may provide cross-NDB binding, similar to the conformational transitions observed during the Wzm-WztN cycle (Spellmon et al. 2022).

Finally, our mutagenesis of the key residues in the Coupling helices (DrrB R121A and DrrC W115A) (see Fig. 4 and Supplementary Figures S2, S7), also validated the proposed coupling mechanism of DrrABC, which we propose is effectuated *via* elements, including CHs, and utilising conserved functional residues, homologous to the type V exporters suggesting similar functional cycle.

Taken together our data, combined with the structural modelling and functional analysis, identifies DrrABC as a unique member of type V ABC-exporter family, which while sharing general ATPase motifs and coupling mechanisms, possesses specialised features, including putative substrate-binding CTDs, essential to its function.

### 3.3 Transport of PGLs in *drr* mutant strains

PGLs share a common chemical core with PDIMs and in our previous studies we observed that the *mmpL7* mutant which is defective in PDIM transport, was also unable to export PGL, though intriguingly, attempts to complement the PGL export phenotype with a plasmid borne copy of *mmpL7* were unsuccessful as repeated analysis of multiple transformants showed a complete loss of PGL biosynthesis. This was because the tested complemented strains all had mutations in the gene encoding FadD22 (a p-hydroxybenzoyl-AMP-ligase priming PGL biosynthesis) abolishing PGL biosynthesis altogether in the complemented strain (Simeone et al. 2010). Our novel mutant strains allowed us for the first time to query the requirement of individual *drr* genes for the transport of the structurally related PGLs. PGL was evident in the outside fraction of all strains except the mutant, which instead showed an intracellular accumulation of PGL (Supplementary Figure S1). However, similar to what was observed in the complemented *mmpL7* mutant (Moolla et al. 2021), the various *drr* complemented strains did not show detectable levels of PGL for the intracellular fraction (Supplementary Figure S1).

## 4 Methods and materials

### 4.1 Bacterial strains and growth condition

For all cloning purposes *Escherichia coli* (*E. coli*) TOP10 (Invitrogen) and HB101 (Stratagene) were grown at 37 °C in Luria Bertani broth or on Luria Bertani agar plates. Liquid cultures of *Mycobacterium bovis *Bacillus Calmette-Guérin (*M. bovis *BCG Pasteur) were grown in Middlebrook 7H9 broth (BD) and supplemented with 0.05 % v/v Tween-80 (Sigma) and 10 % v/v oleic acid-albumin-dextrose-catalase (OADC; BD). For antibiotic selection of *E. coli*, media was supplemented with 100  μg/ml hygromycin or 50 μg/ml kanamycin, while for *M. bovis BCG* media was supplemented with 50  μg/ml hygromycin or 25 μg/ml.

### 4.2 Generation of the *in silico* DrrA, DrrB and DrrC structure

Originally, the homology models for this study were derived using I-TASSER (Yang et al. 2015), alongside Swiss-Model (Waterhouse et al. 2018), however, since the advent of the AI-powered tools AlphaFold2 (Jumper et al, 2021) and, more recently AlphaFold3 (Abramson et al., 2024), these have been superseded. As the AlphaFold2 (AF2) models are freely accessible to all researchers via AlphaFold Protein Structure Database server at EMBL-EBI (https://alphafold.ebi.ac.uk), and represent the state-of-the-art in the field, we have re-rendered all the figures using the latest AF2 models available. Those correspond to the following access codes: DrrA (P9WQL9; AF-P9WQL9-F1-v4), DrrB (P9WG23; AF-P9WG23-F1-v4) and DrrC (P9WG21; AF-P9WG21-F1-v4). However, for consistency, we also provide an overview of our initial modelling routine, as a Supplementary Information, which also provides expanded discussion on the template sequence identities, and as a Supplementary Figure S1 we provide a superposition of the old models with the AF2 models.

For modelling the quaternary assembly of the complete DrrABC complex, manual docking in Coot (Emsley and Cowtan 2004) was performed, guided by the quaternary structures of type V ABC-transporters including the eukaryotic ABCG2 (Orlando and Liao 2020) and ABCA1 exporters (Qian et al. 2017), alongside the prokaryotic floppases TarGH,PDB ID 6JB (Chen et al. 2020) and Wzm-WztN (PDB ID 6 M96; PDB ID 7K2T; PBD ID 6OIH) (Bi et al., 2018, Caffalette et al., 2019, Caffalette and Zimmer, 2021). Additional insight was provided by the apo- and O-antigen-occupied structures of the *Aquifex aeolicus* Wzm-WztN (8DKU, 8DKY, 8DL0, 8DN8, 8DNC, 8DNE, 8DOU), as they present a resolved CTD (Spellmon et al. 2022).

Structure comparison and model quality analysis were performed using the Pymol (The Pymol Molecular Graphics System, version 1.8, Schrödinger, LLC) and Coot package (Emsley and Cowtan, 2004). Where relevant, the structural superpositions and RSMD calculations were calculated using the SSM Superpose function as implemented within Coot. Pairwise and multiple sequence alignment has been performed using MAFFT (Katoh and Standley, 2013) and resulting alignments were used for identity and similarity calculations using Ident and Sim (Stothard, 2000). Multiple sequence alignments were visualized using Espript3 (https://espript.ibcp.fr) (Robert and Gouet 2014).

### 4.4 *drrABC* mutant generation in *m. bovis *BCG

The entire *drr* operon of *M. bovis *BCG containing genes *drrA*, *drrB* and *drrC* were disrupted by replacing these genes with a hygromycin resistance gene using specialized transduction as described by earlier (Bardarov et al., 2002, Larsen et al., 2007). Briefly an allelic exchange substrate (AES) construct was created by cloning in-frame 1 kb PCR fragments upstream of *drrA* (using primer pair UF: 5′- TTTTTTTTCAC**AAA***GTG*GCCCTTCCCAACGCGCATACC- 3′ and UR: 5′- TTTTTTTTCAC**TTC**GTGGCGGCGCACCTTGAAACTCAC- 3′ where restriction sites and overhangs are denoted by underlined and bold sequences) and downstream of *drrC* (using primer pair DF: 5′- TTTTTTTTCAC**AGA**GTGGTCCTCTCTCCGATGATCGGG- 3′ and DR: 5′- TTTTTTTTCAC**CTT**GTGCTGCCACTCGGTCAGCAGGAT- 3′ where restriction sites and overhangs are denoted by underlined and bold sequences) from H37Rv genomic DNA, into p0004S, flanking a hygromycin resistance cassette and verified by DNA sequencing. The AES construct was packaged into the temperature sensitive mycobacteriophage phAE159 resulting in phΔ*drrABC*, which was used to transduce wild type *M. bovis *BCG (Larsen et al. 2007). Hygromycin resistant transductants were selected and screened by PCR and verified by whole genome sequencing. Only one transductant was designated Δ*drrABC* and used for all further studies.

### 4.5 Functional complementation

The *drrABC* mutant was complemented with the wild type gene *drrABC* amplified from H37Rv genomic DNA using the ABC forward primer 5′- CATGCATG TCTAGA AAGTCCGTTCCGCACTACGGA- 3′ and ABC reverse primer 5′- CATGCATG AAGCTT CTGGGTGGGTTTCCAAGAGGG- 3′ (restriction sites are underlined). This fragment was cloned into pMV306 using *Xba*I and *Hind*III to create pMV306::*drrABC* which was then verified by DNA sequencing. The recombinant plasmid pMV306::*drrABC* was introduced to the *drrABC* mutant by electroporation to generate the strain *drrABC* C. The *drrABC* mutant was also used to generate strains by complementation that have a loss of individual genes (*drrA*, *drrB* and *drrC*) (Supplementary Figure 1) in two ways.

The first technique used involved three-way cloning which was only successful in generating the pMV306*drrAB* plasmid. Two fragments were amplified using the ABC forward primer as stated previously with the AB reverse primer 5′- CATGCATGGAATTCAATGCTGTAGAGCCCGCTGTC- 3′ and the AB forward primer 5′- CATGCATGGAATTCTGGATTCAGCCGTTCGTCGCC- 3′ with the ABC reverse primer as mentioned before (restriction sites are underlined) and cloned into pMV306 simultaneously. For the generation of pMV306*drrBC* and pMV306*drrAC* another technique involved the use of the site directed mutagenesis kit from NEB, where the sequence verified recombinant plasmid pMV306::*drrABC* served as a template for selective amplification using primer pairs AC forward primer 5′-GAATTCAAGCTGTTTCCGCACTGGATCCAT- 3′ and AC reverse primer 5′- CACCGTGGTGAGTACTTCACCGTT- 3′ and BC forward primer 5′- GAATTCGACTCAGACCGCATTACGATGCCG- 3′ and BC reverse primer 5′- CGGTTCGGAAACAACATCGTAGCC- 3′ respectively (primers were designed using the website: https://nebasechanger.neb.com/). All the complementation plasmids- pMV306*drrBC*, pMV306*drrAC* and pMV306*drrAB*, were sequence verified and electroporated into the *drrABC* mutant strain and then kanamycin and hygromycin resistant transformants were selected and screened by PCR. Only one positive transformant of each strain was designated as Δ*drrA*, Δ*drrB* and Δ*drrC*.

### 4.6 PDIM/PGL analysis

Strains listed in table and the wild type strain were grown in 10 ml liquid media until logarithmic phase (OD _600_ of ∼ 0.6) then 0.2  µCi/mL [^14^C] sodium propionate (specific activity 50–60  mCi m/mol; American Radiolabelled Chemicals) was added to the cultures (10  ml) and allowed to incubate for 24 h, shaking. Cultures were harvested by centrifugation at 3500 rpm for 10 min where the spent culture filtrate was filtered (0.22 µm) for lipid extraction while the cell pellets were dried on a heating block at 50 °C under nitrogen. The filtered spent culture filtrate was extracted with equal amounts of petroleum ether (60–80 °C) and placed on a rotator for 1 h, then centrifuged at 4000 rpm for 10 min. The upper layer was transferred to a clean tube and the process was repeated. The pooled culture filtrate extracts were dried on a heating block (at 50 °C) under nitrogen. To the dried biomass 2 ml of methanol: 0.3 % NaCl (10:1, v/v) followed by 2 ml of petroleum ether (60–80 °C) were added and placed on a rotator for 15 min and centrifuged at 3500 rpm for 10 min. The upper layer was transferred to a clean tube and the process repeated to extract the remaining inside apolar lipids. The accumulated inside apolar extracts were dried on a heating block (at 50 °C) under nitrogen. Dried culture filtrate and inside apolar extracts represent the outside and inside of the cell membrane and were resuspended in 200  µl chloroform: methanol (2:1, v/v). One-dimensional thin-layer chromatography (1D-TLC) was used to analyse PDIM and PGLs. Equal radioactivity about 20 000 counts per min (cpm) from each sample was loaded onto a silica gel 60 F_254_ plate (Merck), and resolved using chloroform: methanol (9:1, v/v) to resolve PDIM and for chloroform: methanol (95:5, v/v) PGLs and were visualized after 72 hrs exposure to Kodak X-Omat AR film by autoradiography.

### 4.7 RT-PCR

About 50 ml of liquid cultures of wild type *M. bovis* BCG, *drrABC* mutant and the three single *drr* mutants each, were grown until mid logarithmic phase (A_600_ of 0.8) and the total RNA was extracted from the pellet using the protocol as described earlier (Mangan et al. 1997). The RNA was treated with Turbo DNA free kit DNase (Invitrogen) to remove any traces of genomic DNA and an inactivation resin to remove DNase and divalent cations from the RNA. The purified RNA samples (2 ug each) were converted to cDNA using random hexamers and the SuperScript™ III Reverse Transcriptase kit from Invitrogen as per manufacturer’s instructions. A set of no reverse transcriptase reactions were used as controls. The *sigA* gene was used a control for constitutive expression. PCR was carried out as per the manufacturer’s instructions using the reagents provided in the Q5 polymerase kit (NEB) and PCR cycling conditions were set at 35 cycles. The transcripts (20 µl each) were analysed on a 2 % agarose gel. The primers used in this study were sigA-f (TTCGCGCCTACCTCAAACAG); sigA-r (AGGTTGGCTTCCAGCAGATG); drrA-f (GCTGGCTACGATGTTGTTTCC); drrA-r (GGCATGTACGAGGCTGAATTG); drrB-f (ACAGATGTGGGTGCTCTATCG); drrB-r (GGCGTGATGTATTGCCCTAAG); drrC-f (GAGCTTGCACCCACACGTTTG) drrC-r (AATGCTGTAGAGCCCGCTGTC).

### CRediT authorship contribution statement

**Nabiela Moolla:** Writing – original draft, Validation, Methodology, Investigation, Formal analysis, Data curation, Conceptualization. **Helen Weaver:** Writing – original draft, Methodology, Investigation, Formal analysis, Data curation, Conceptualization. **Rebeca Bailo:** Methodology, Investigation, Formal analysis, Data curation, Conceptualization. **Albel Singh:** Methodology, Investigation. **Vassiliy N. Bavro:** Writing – review & editing, Writing – original draft, Validation, Methodology, Investigation, Formal analysis, Data curation, Conceptualization. **Apoorva Bhatt:** Writing – review & editing, Writing – original draft, Funding acquisition, Formal analysis, Conceptualization.

## Declaration of competing interest

The authors declare the following financial interests/personal relationships which may be considered as potential competing interests: Apoorva Bhatt reports financial support was provided by UK Research and Innovation Medical Research Council. If there are other authors, they declare that they have no known competing financial interests or personal relationships that could have appeared to influence the work reported in this paper.
