# Supplementary material for: The role of ABC transporter DrrABC in the export of PDIM in Mycobacterium tuberculosis
Source: Cell Surf. 2024 Oct 15;12:100132. doi: 10.1016/j.tcsw.2024.100132 (PMC11539658; doi:10.1016/j.tcsw.2024.100132)

Supplementary Figure S1.

Comparison of the I-TASSER (blue) vs AlphaFold2 (red) models of DrrABC assembly shows close convergence, apart from the CTD, which could not be modelled reliably using I-TASSER due to lack of close homology templates. The view is 180 degrees rotated along the vertical axis relative to the view from Figure 4.

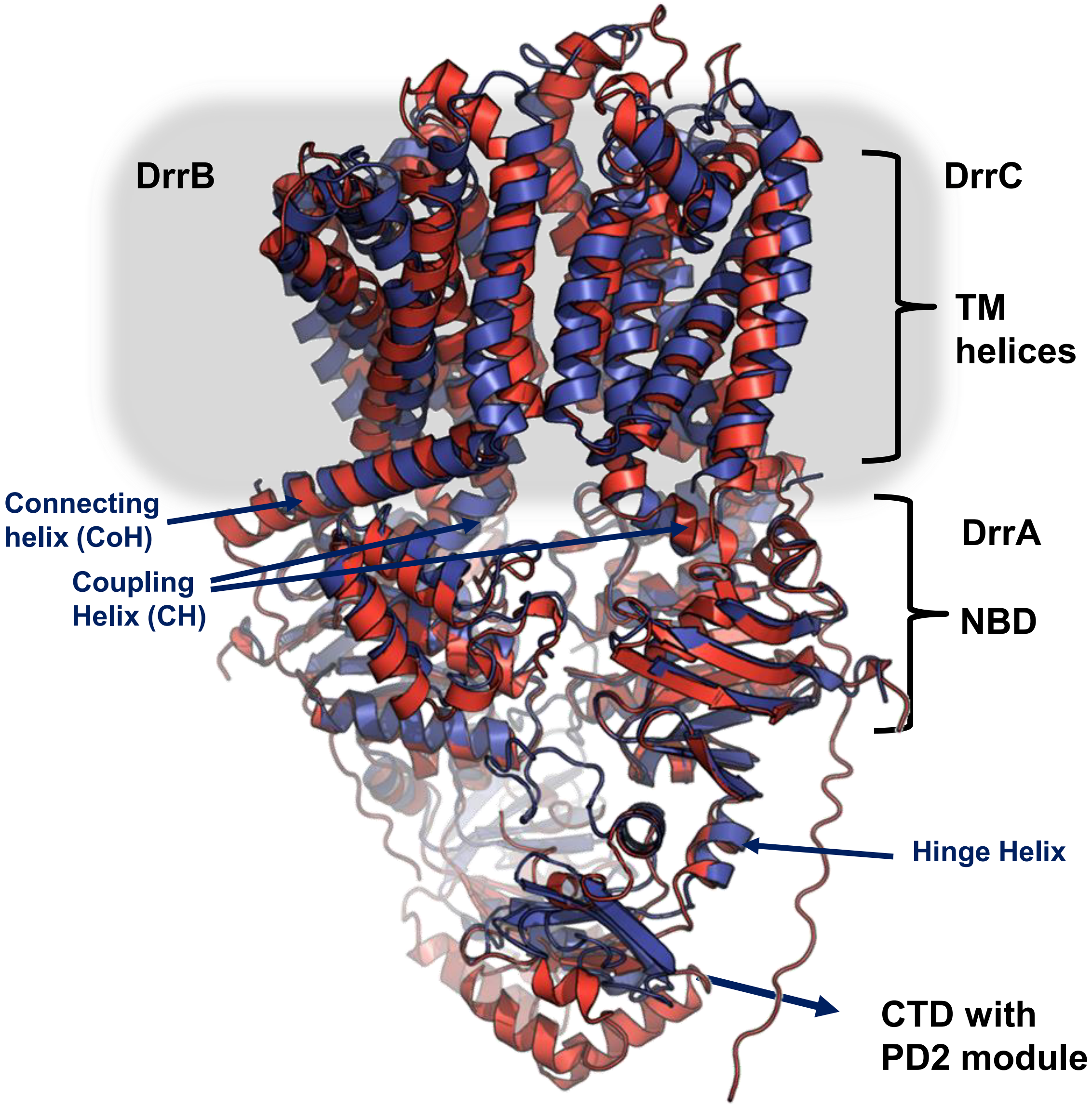

Supplementary Figure S2.

Comparison of the experimental structure of Wzm-WztN in a substrate and nucleotide free state, 8DL0.pdb (top) vs the AlphaFold2 model of the DrrABC transporter, showing the close overall organisation and the analogous position of the CBD and CTD respectively. Location of the key mutations introduced into DrrABC in this work are also shown.

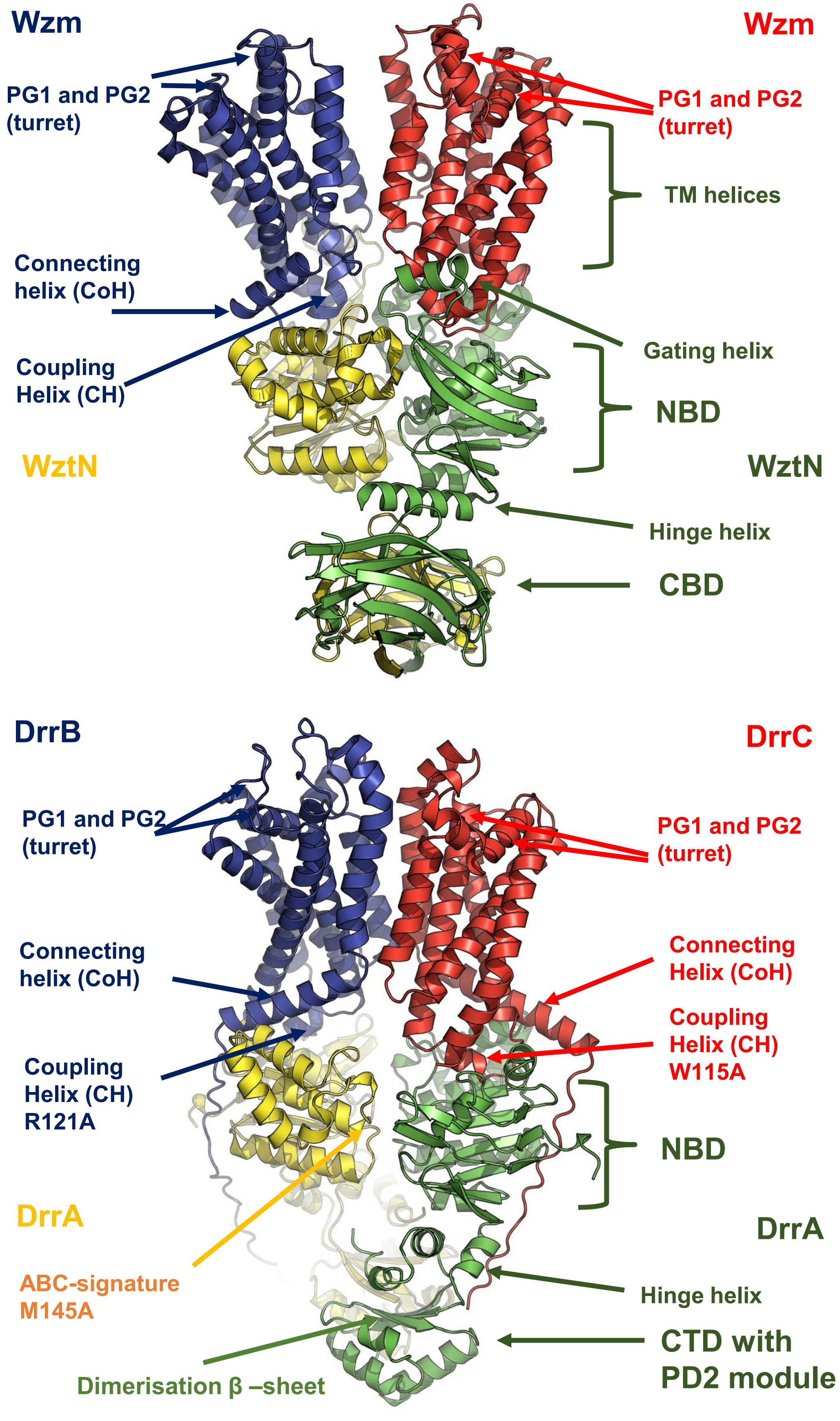

Supplementary Figure S3.

S3A. An AlphaFold3 model of the dimer of DrrA, lateral view from the membrane and rotated 90 degrees. The two protomers are shown in gold and green. The PD2 module is highlighted with a yellow shaded area.

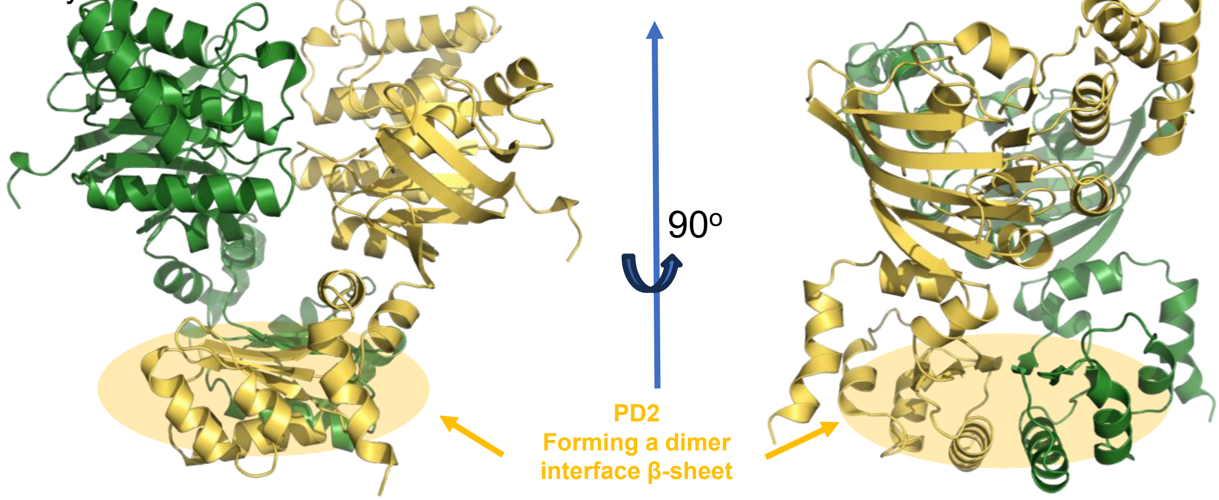

S3B. A different representation of the DrrA dimer showing the domain organisation and key structural elements discussed in the text. One subunit is shown in grey, the other in rainbow (from N-terminus in blue to C-terminus in red).

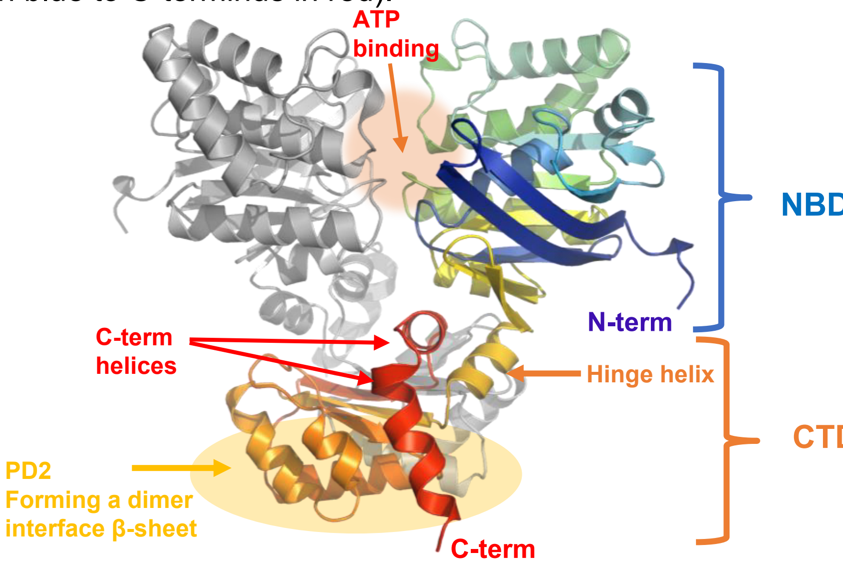

S3C. The experimental structure of the closest structural relative of DrrA with known structure, the TM_1403 transporter from *Thermotoga maritima,* highlighting identical dimerization and the location of the ATP-binding pockets occupied by ATP (the back one is indicated with a dotted line) as seen in 4YER.pdb.

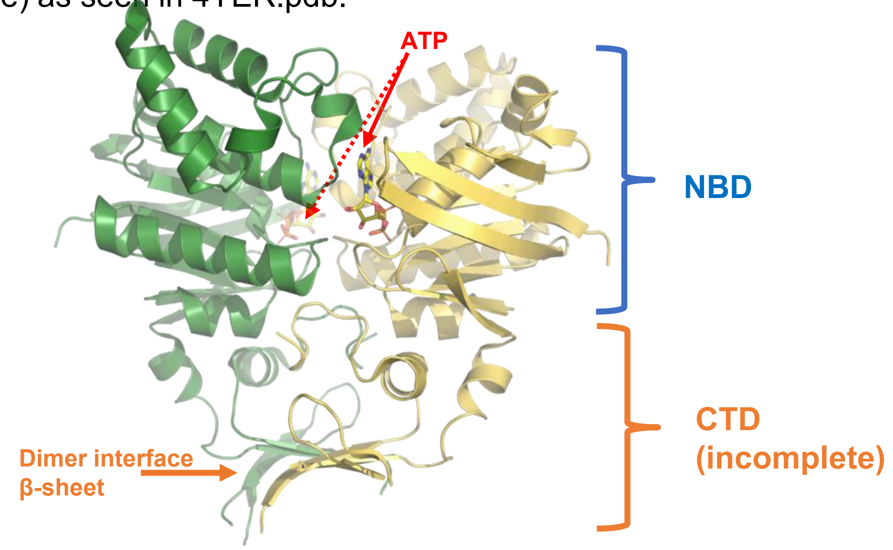

Supplementary Figure S4.

A visualisation of the predicted effect of the different DrrA truncation constructs based on the AlphaFold3 modelling. Left to right: Full length DrrA, the DrrA lacking just the PD2 module (ΔG232-P305) or the entire CTD (ΔA224-R331). The conserved NBD-core of DrrA or its packing is not expected to undergo significant changes.

Area magnified in the Supplementary figure S5 below is shown as an orange box.

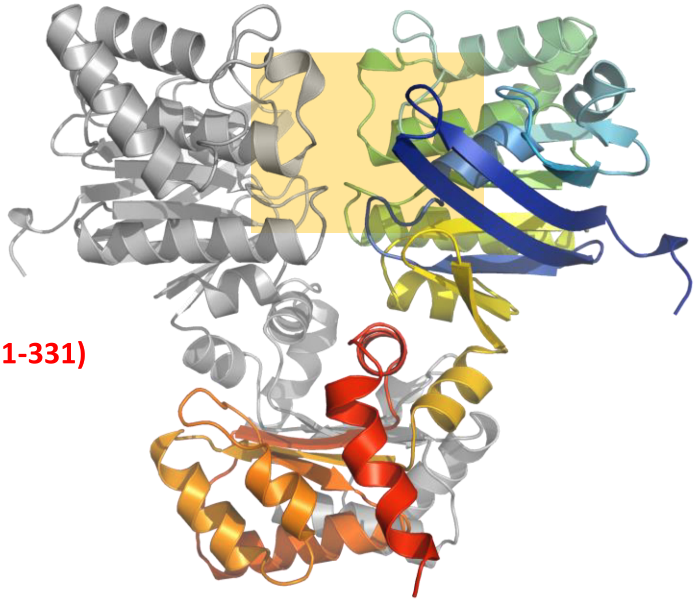

DrrA full length (1-331)

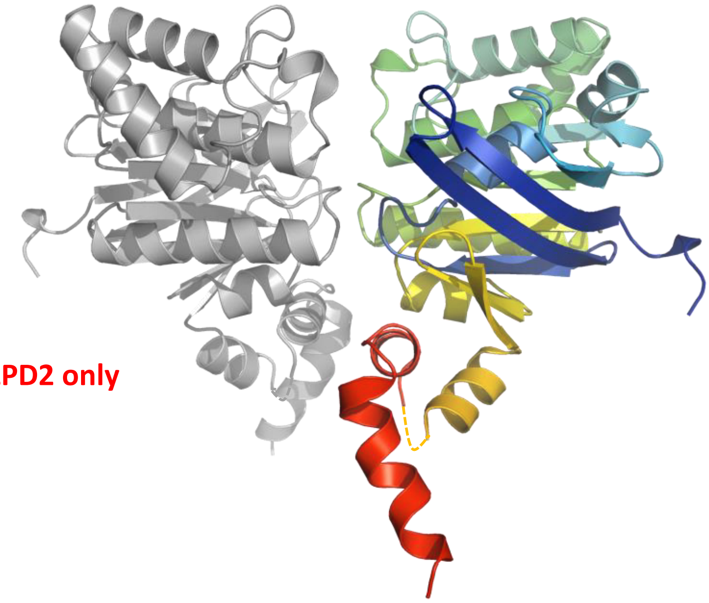

ΔG232-P305 – ΔPD2 only

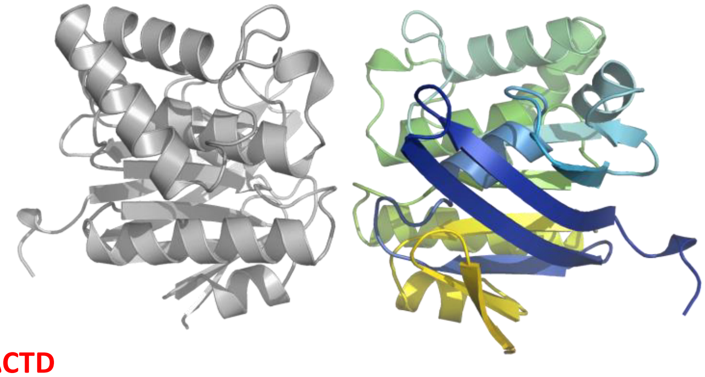

ΔA224-R311 – ΔCTD

Supplementary Figure S5.

A close-up of the predicted ATP-binding site in DrrA and comparison with other related transporters of known structure highlights conservation of the critical residues involved in ATP binding. **S5A** – AlphaFold3 model of DrrA dimer. **S5B** – experimental structure of the ATP-bound form of TM_1403 from *Thermotoga maritima,* sharing 37% sequence identity with DrrA, based on the 4YER1.pdb. **S5C** – experimental structure of the ATP-bound form of WztN based on the 7K2T.pdb. The different sequence elements discussed in the text are coloured as follows: *Walker A* – cyan; *Walker B* – red ; *ABC-signature* (aka *C-motif*) – magenta. Several key residues are labelled, including the mutated M145A.The hydrophobic residue -A132 (DrrA) forming the top of the hydrophobic cavity for M140/M146 is shown in orange and corresponds to F128 (TM_1403) / F134 (WztN) respectively.

S5A

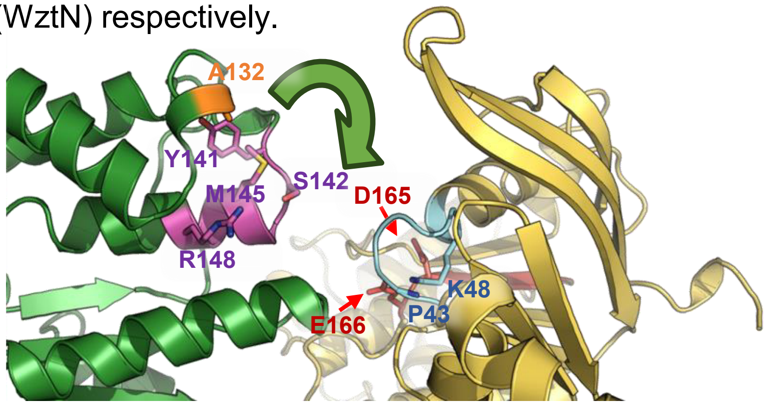

S5B

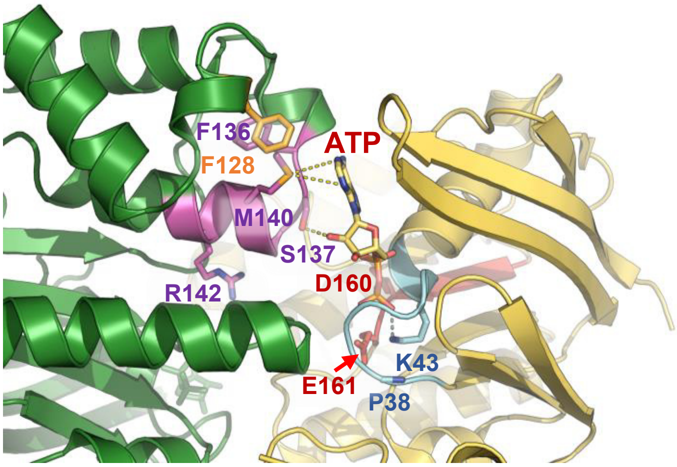

S5C

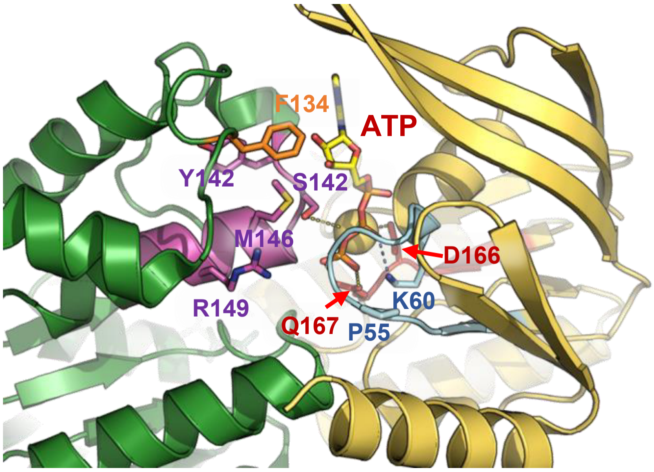

Supplementary Figure S6.

S6A. Pairwise alignment of the DrrA and Tm_1403 showing the conserved sequence boxes involved in the ATP-binding and key structure elements discussed in the text.

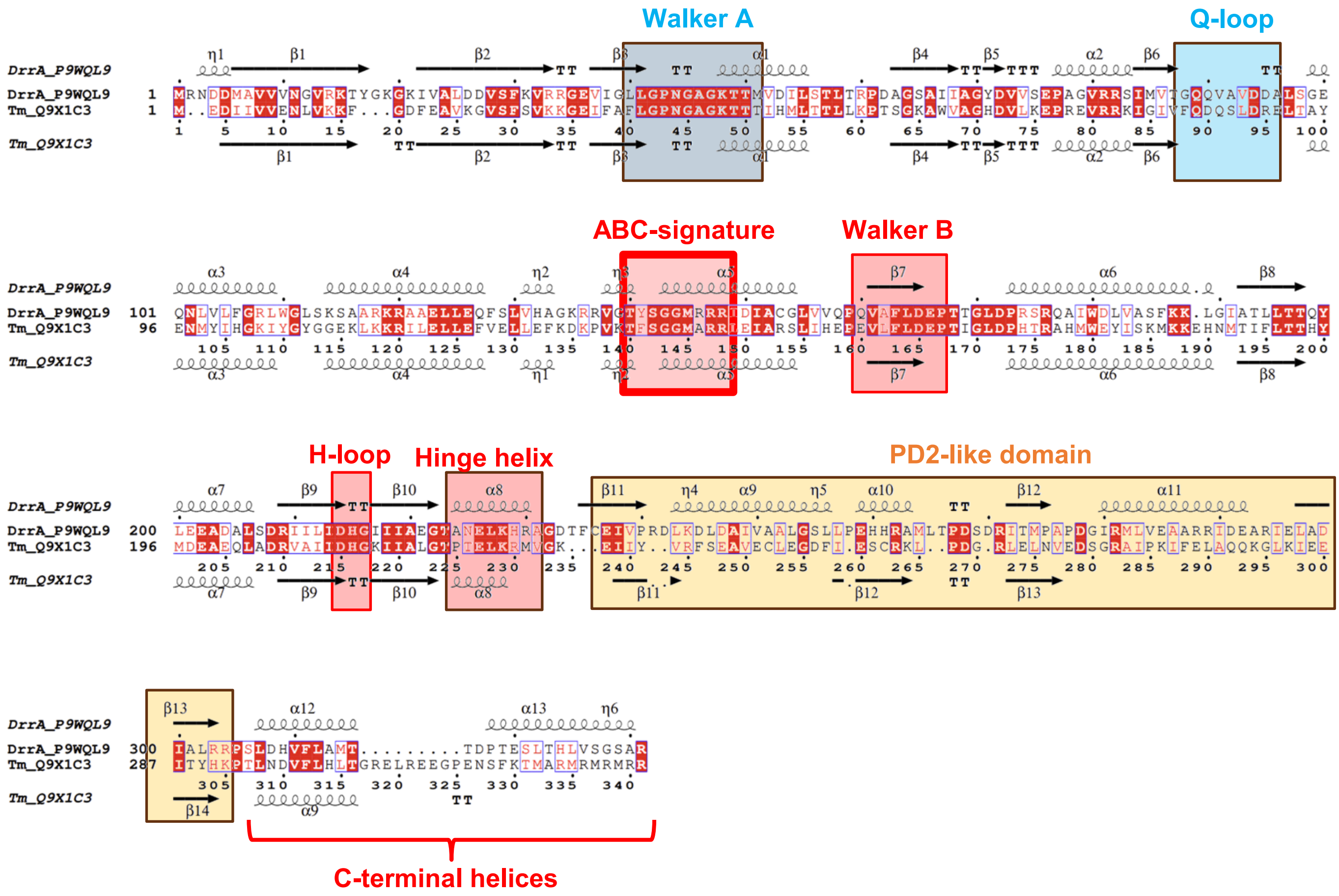

Supplementary Figure S6.

S6B. Pairwise alignment of the DrrA, Tm_1403 and WztN showing the conservation of the NDB domain topology and the differences in the CTD/CBD respectively as discussed in the text.

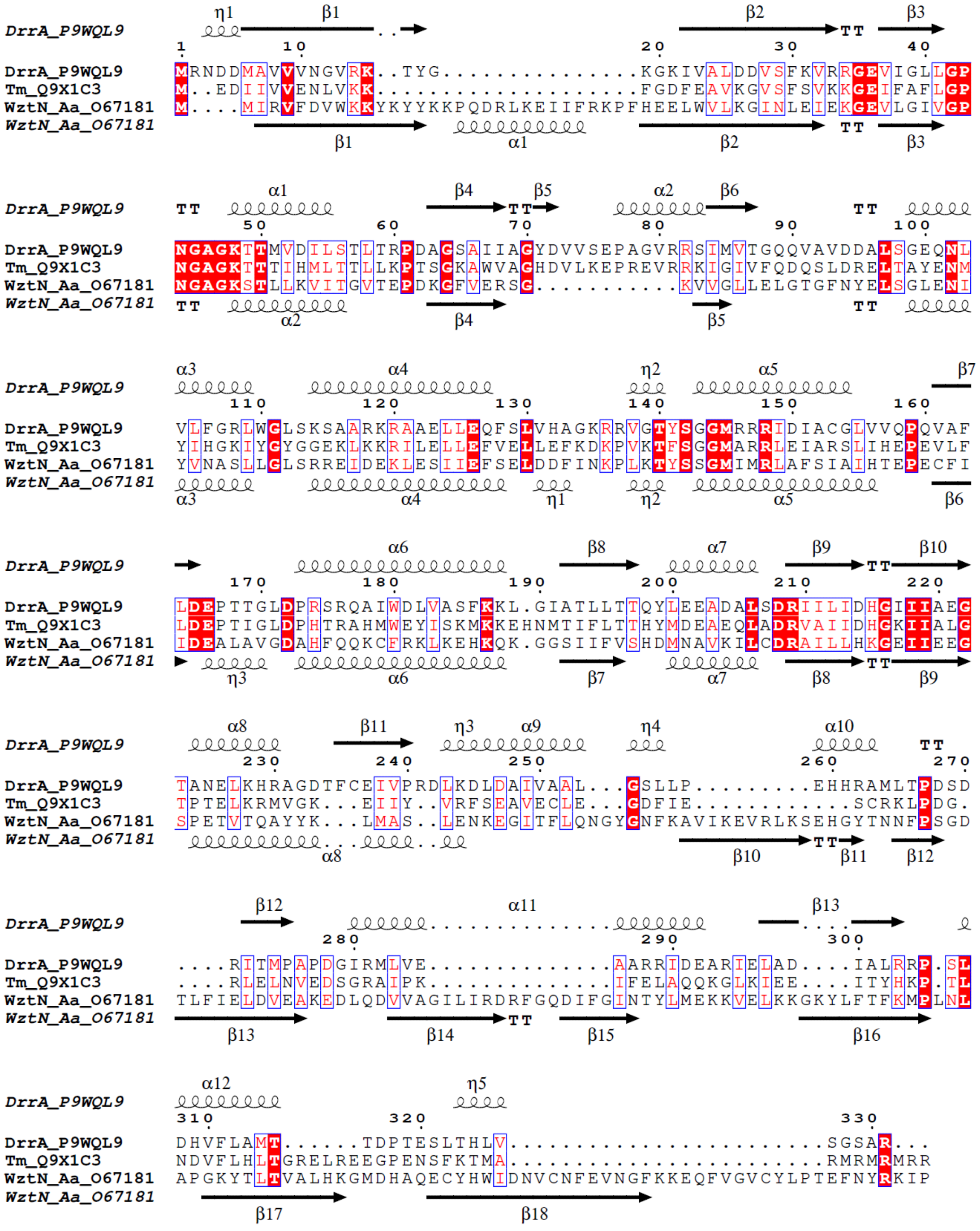

Supplementary Figure S7.

S6B. Pairwise alignment of the Wzm, DrrB and DrrC showing the conservation of overall topology, highlighting the key structural elements discussed in the text.

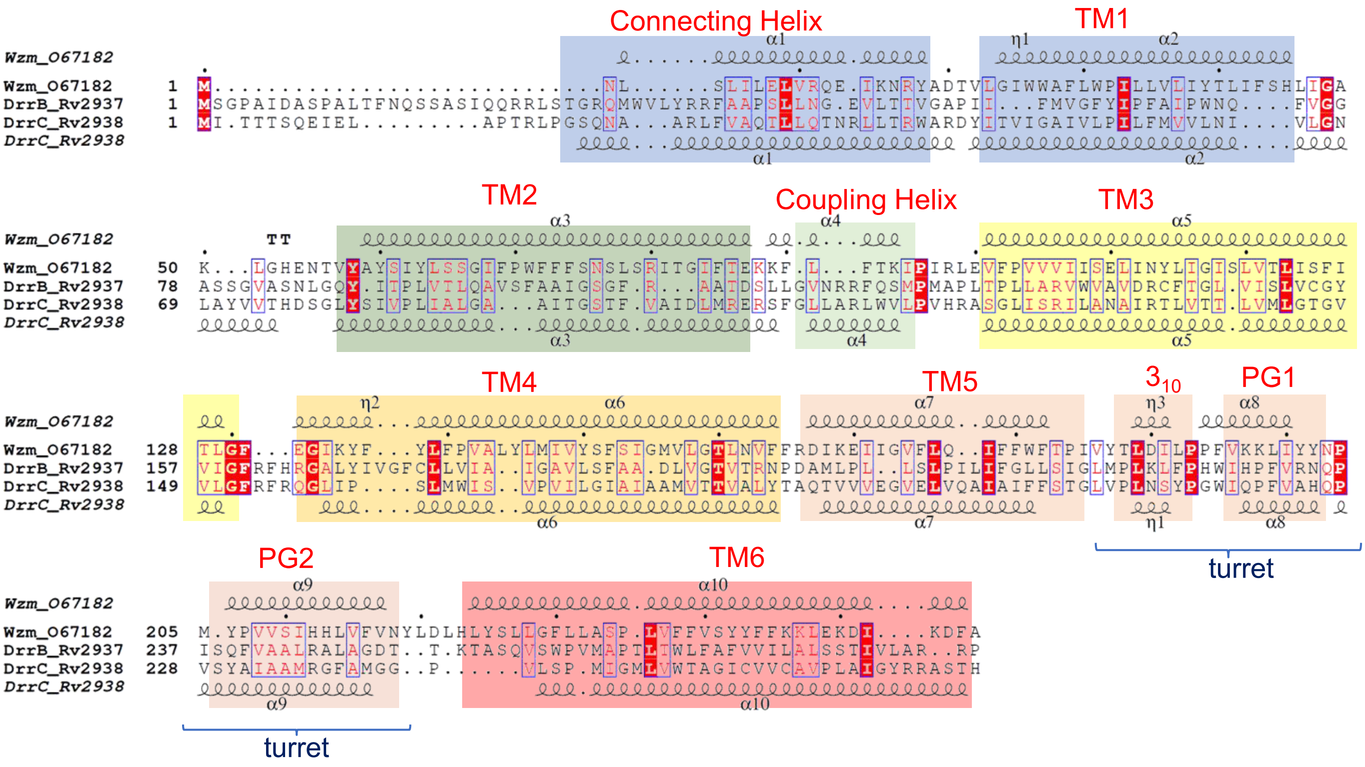

Supplement: Supplementary Data 1 [file mmc1.pdf]
