# Supplementary material for: The role of ABC transporter DrrABC in the export of PDIM in Mycobacterium tuberculosis
Source: Cell Surf. 2024 Oct 15;12:100132. doi: 10.1016/j.tcsw.2024.100132 (PMC11539658; doi:10.1016/j.tcsw.2024.100132)
Supplement: Supplementary Data 2 [file mmc2.pdf]

Supplementary information:

As mentioned in the main text, originally, the homology modelling of the Drr proteins has been performed using I-TASSER (Yang et al. 2015), alongside Swiss-Model (Waterhouse et al. 2018).

To identify suitable structural templates for homology modelling of the DrrABC, we used BLASTp against the PDB database, and then run I-TASSER in template-specific mode (Yang et al. 2015), alongside Swiss-Model (Waterhouse et al. 2018), with manual selection of templates using the top scoring sequences. The following specific templates were used. DrrA: The ABC-transporter TM_1403 from *Thermotoga maritima* MSB8 presenting 35.97% identity (4YER.PDB; Joint Center for Structural Genomics (JCSG); 2015); alongside the human ABCA1 with 29% identity (5XJY.pdb) (Qian et al. 2017), and the retinal-specific phospholipid-transporting ATPase ABCA4 7LKZ.pdb (Liu, Lee, and Chen 2021). DrrB: Eukaryotic ABC transporters Pdr5 from *Saccharomyces cerevisiae* (16.74% identify; PDB ID 7P04.PDB) (Harris et al. 2021); as well as the ABCG2 exporter (10.67% identity; 7OJI.pdb) (Yu et al. 2021); alongside the prokaryotic teichoic acid ABC transporter TarGH (6JBH.pdb) (Chen et al. 2020), and the O-antigen transporter Wzm-Wzt (PDB ID 6M96; PDB ID 7K2T; PBD ID 6OIH) (Bi et al. 2018; Caffalette et al. 2019; Caffalette and Zimmer 2021). DrrC: The template with the highest identity corresponded to the ABCG2 (13.64% identity; 6VXI.PDB) (Orlando and Liao 2020); alongside the aforementioned Wzm-Wzt flippase (11.62% sequence identity; 6OIH.pdb) (Bi et al. 2018), and TarG (11.42% identity) (Chen et al. 2020). In addition, 7R88.pdb (11.16% identity); belonging to the human ABC-transporter cassette sub-family G member 5 (ABCG5) (Sun et al. 2021). We built models based on all of the above templates, while additional models (accession codes P9WQL9 (DRRA_MYCTU); P9WG23 (DRRB_MYCTU); P9WG21 (DRRC_MYCTU) for DrrA, DrrB and DrrC respectively), were obtained based on modelling provided by AlphaFold2 (Jumper et al. 2021) from the AlphaFold Protein Structure Database server at EMBL-EBI (https://alphafold.ebi.ac.uk) upon its release. Due to the higher general quality of the latter models, we have used them for the illustrative purposes, however they show close convergence to the I-TASSER and Swiss-Model (See Supplementary Figure 1), and all of the initial conclusions derived from the earlier models appear equally supported by the newer AlphaFold ones.

References

Bi, Y., E. Mann, C. Whitfield, and J. Zimmer. 2018. 'Architecture of a channel-forming O-antigen polysaccharide ABC transporter', *Nature*, 553: 361-65.

Caffalette, C. A., R. A. Corey, M. S. P. Sansom, P. J. Stansfeld, and J. Zimmer. 2019. 'A lipid gating mechanism for the channel-forming O antigen ABC transporter', *Nat Commun*, 10: 824.

Caffalette, C. A., and J. Zimmer. 2021. 'Cryo-EM structure of the full-length WzmWzt ABC transporter required for lipid-linked O antigen transport', *Proc Natl Acad Sci U S A*, 118.

Chen, L., W. T. Hou, T. Fan, B. Liu, T. Pan, Y. H. Li, Y. L. Jiang, W. Wen, Z. P. Chen, L. Sun, C. Z. Zhou, and Y. Chen. 2020. 'Cryo-electron Microscopy Structure and Transport Mechanism of a Wall Teichoic Acid ABC Transporter', *mBio*, 11.

Harris, A., M. Wagner, D. Du, S. Raschka, L. M. Nentwig, H. Gohlke, S. H. J. Smits, B. F. Luisi, and L. Schmitt. 2021. 'Structure and efflux mechanism of the yeast pleiotropic drug resistance transporter Pdr5', *Nat Commun*, 12: 5254.

Jumper, J., R. Evans, A. Pritzel, T. Green, M. Figurnov, O. Ronneberger, K. Tunyasuvunakool, R. Bates, A. Zidek, A. Potapenko, A. Bridgland, C. Meyer, S. A. A. Kohl, A. J. Ballard, A. Cowie, B.

Romera-Paredes, S. Nikolov, R. Jain, J. Adler, T. Back, S. Petersen, D. Reiman, E. Clancy, M. Zielinski, M. Steinegger, M. Pacholska, T. Berghammer, S. Bodenstein, D. Silver, O. Vinyals, A. W. Senior, K. Kavukcuoglu, P. Kohli, and D. Hassabis. 2021. 'Highly accurate protein structure prediction with AlphaFold', *Nature*, 596: 583-89.

Liu, F., J. Lee, and J. Chen. 2021. 'Molecular structures of the eukaryotic retinal importer ABCA4', *Elife*, 10.

Orlando, B. J., and M. Liao. 2020. 'ABCG2 transports anticancer drugs via a closed-to-open switch', *Nat Commun*, 11: 2264.

Qian, H., X. Zhao, P. Cao, J. Lei, N. Yan, and X. Gong. 2017. 'Structure of the Human Lipid Exporter ABCA1', *Cell*, 169: 1228-39 e10.

Sun, Y., J. Wang, T. Long, X. Qi, L. Donnelly, N. Elghobashi-Meinhardt, L. Esparza, J. C. Cohen, X. S. Xie, H. H. Hobbs, and X. Li. 2021. 'Molecular basis of cholesterol efflux via ABCG subfamily transporters', *Proc Natl Acad Sci U S A*, 118.

Waterhouse, A., M. Bertoni, S. Bienert, G. Studer, G. Tauriello, R. Gumienny, F. T. Heer, T. A. P. de Beer, C. Rempfer, L. Bordoli, R. Lepore, and T. Schwede. 2018. 'SWISS-MODEL: homology modelling of protein structures and complexes', *Nucleic Acids Res*, 46: W296-W303.

Yang, J., R. Yan, A. Roy, D. Xu, J. Poisson, and Y. Zhang. 2015. 'The I-TASSER Suite: protein structure and function prediction', *Nat Methods*, 12: 7-8.

Yu, Q., D. Ni, J. Kowal, I. Manolaridis, S. M. Jackson, H. Stahlberg, and K. P. Locher. 2021. 'Structures of ABCG2 under turnover conditions reveal a key step in the drug transport mechanism', *Nat Commun*, 12: 4376.
